# Boosting therapeutic potency of antibodies by taming Fc domain functions

**DOI:** 10.1038/s12276-019-0345-9

**Published:** 2019-11-18

**Authors:** Tae Hyun Kang, Sang Taek Jung

**Affiliations:** 1 Biopharmaceutical Chemistry Major, School of Applied Chemistry, Kookmin University, Seongbuk-gu, Seoul, 02707 Republic of Korea; 2 Department of Biomedical Sciences, Graduate School of Medicine, Korea University, Seoul, 02841 Republic of Korea

**Keywords:** Drug development, Antibody therapy, Drug development, Antibody therapy

## Abstract

Monoclonal antibodies (mAbs) are one of the most widely used drug platforms for infectious diseases or cancer therapeutics because they selectively target pathogens, infectious cells, cancerous cells, and even immune cells. In this way, they mediate the elimination of target molecules and cells with fewer side effects than other therapeutic modalities. In particular, cancer therapeutic mAbs can recognize cell-surface proteins on target cells and then kill the targeted cells by multiple mechanisms that are dependent upon a fragment crystallizable (Fc) domain interacting with effector Fc gamma receptors, including antibody-dependent cell-mediated cytotoxicity and antibody-dependent cell-mediated phagocytosis. Extensive engineering efforts have been made toward tuning Fc functions by either reinforcing (e.g. for targeted therapy) or disabling (e.g. for immune checkpoint blockade therapy) effector functions and prolonging the serum half-lives of antibodies, as necessary. In this report, we review Fc engineering efforts to improve therapeutic potency, and propose future antibody engineering directions that can fulfill unmet medical needs.

## Monoclonal antibodies: Engineering immunity

Fine-tuning the function of monoclonal antibodies (mAbs) holds promise for developing new therapeutic agents. Antibodies bind to pathogens or cancer cells, flagging them with Fc (fragment crystallizable) domain for destruction by the immune system. mAbs attached only to specific target cells enable lower side effect than other conventional drugs. Sang Taek Jung at Korea University and Tae Hyun Kang at Kookmin University, both in Seoul, reviewed recent developments in engineering therapeutic potency of mAbs. They report that mAbs can be engineered to activate effective immune cell types to treat a particular disease. Engineering can also increase mAbs’ persistence in the blood, enabling less frequent administration. Antibodies engineered to bind to two different antigens at once can also improve therapeutic efficacy. Applying these techniques could help developing new treatments against cancer, and infectious and autoimmune diseases.

## Introduction

Thirty-one new monoclonal antibodies (mAbs) have been approved by the Food and Drug Administration (FDA) in the USA and the European Medicines Agency in the EU since 2013, to constitute a total of 57 mAbs used in clinics by the end of 2017^1^. Several financial reports revealed that the antibody market had exceeded 98 billion US dollars in sales by December 2017, with an annual growth rate of 18.3% and a predicted valuation of 137–200 billion US dollars by 2022^1^. The antibody market is dominated by seven companies, with 87% of the total market sales out of 22 active companies led by Genentech, a member of the Roche group, with a 31% market share for 11 approved antibody molecules. In terms of disease indications, as of December 2017 15 out of 57 mAbs are approved for cancer therapy, the number one disease target, and 12 mAbs are used in hematology.

Among the five isotypes of immunoglobulins (Igs), which are IgG, IgA, IgM, IgD, and IgE, IgG comprises the majority, representing 60% of total serum Igs in humans. All FDA-approved therapeutic Igs belong to the IgG class^2^. The endogenous IgG molecule is composed of two identical fragment antigen binding (Fab) domains and one fragment crystallizable (Fc) domain that make it multivalent and multifunctional. The two Fab fragments each consist of a heterodimer of a light chain and the N-terminal part of the heavy chain, whereas the C-terminal half of the two heavy chains dimerizes to form the Fc fragment of the IgG antibody. The N-terminal domains of the Fab fragment are the variable domains (V_L_ and V_H_) that are responsible for antigen recognition, whereas the C-terminal part of the heavy chains compose the Fc fragment that is responsible for humoral and cellular effector functions. The two Fabs and the Fc are connected by the hinge region, which facilitates the spatial alignment of the three moieties for binding to antigens and effector ligands. The native full-length mAb, recognizing one epitope, comprises 53 out of the total available mAbs in clinics as of December 2017^1^.

Antibody Fc domains are responsible for function in antibodies and Fab domains are responsible for targeting. Thus, Fc engineering stands for engineering functions of antibodies, which are effector functions, such as antibody-dependent cellular cytotoxicity (ADCC) and antibody-dependent cellular phagocytosis (ADCP), and controlling serum half-life. In this article, we discuss (i) Fc receptors, such as Fcγ receptors (FcγRs) and neonatal Fc receptor (FcRn); (ii) Fc engineering modulating binding capacity to FcγRs and FcRn; and (iii) other Fc engineering efforts, such as creating monovalency or bispecificity for improving therapeutic potency.

## Fc receptors: Fcγ receptors (FcγRs) and neonatal Fc receptor (FcRn)

### The Fcγ receptor IIIa

**Fig. 1:**
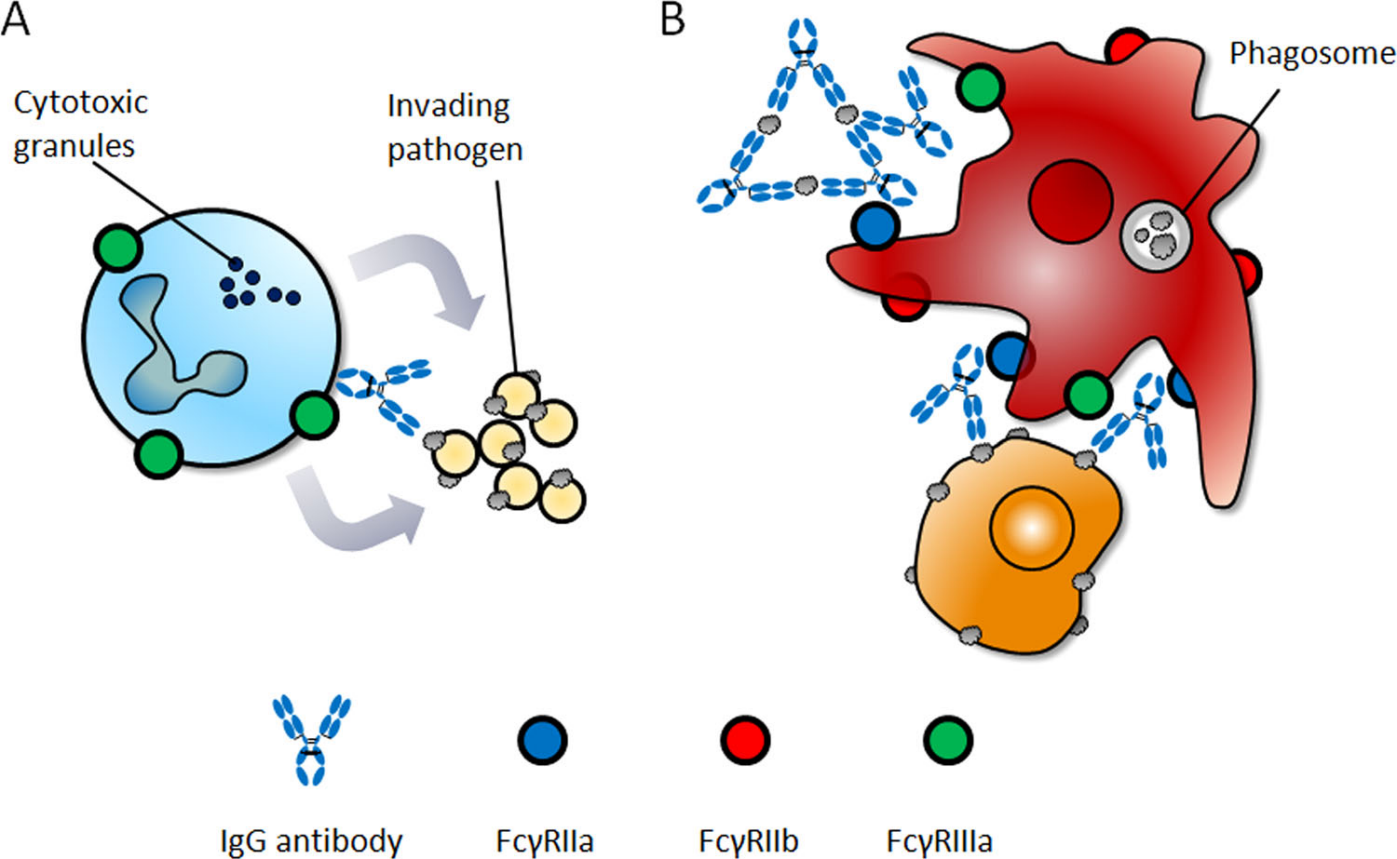
The effector functions of immunoglobulin G antibodies. Human natural killer cells, which only express FcγRIIIa, activate antibody-dependent cellular cytotoxicity via FcγRIIIa (**a**). Macrophages are one of the main contributing lymphocytes for antibody-dependent cellular phagocytosis activity, which is known to be triggered by FcγRIIa intracellular signaling (**b**).

A family of receptors that recognize the Fc domain of IgG molecules is known as the FcγRs family. These receptors are expressed on the surfaces of immune effector cells, and upon being cross-linked by the IgG Fc domain, they induce downstream cellular processes, which affect innate and adaptive immunity. Among the FcγRs, which are highly homologous throughout mammalian species^3^, FcγRIIIa is a key surface receptor in terms of its contribution to ADCC activity (Fig. 1a). Typically, FcγRIIIa is found on the surfaces of natural killer (NK) cells, macrophages, monocytes, mast cells, eosinophils, and dendritic cells. However, it is the only FcγR expressed by NK cells^4,5^. FcγRIIIa is a transmembrane receptor containing two extracellular domains and a cytoplasmic tail, with medium affinity for IgG (*K*_*D*_ *=* 2 × 10^−7^ M)^6^. In FcγRIIIa, an immunoreceptor tyrosine-based activation motif (ITAM) sequence is present in the intracellular region of the associated γ chain or ζ chain. The function of this receptor is dependent either on its γ-chain on monocytes and macrophages or on γ- or ζ-chains on NK cells. Humans express two FcγRIIIa allotypes that differ in a single amino acid at position 158; the residue can be either valine (V) or phenylalanine (F), whereby the isoform with V at position 158 has high affinity for the Fc domain of IgG1, and the one with F at position 158 has low affinity. This FcγRIIIa polymorphism has been shown to contribute to clinical responses of IgG1 therapeutic antibodies^7–10^. Engagement of the high-affinity FcγRIIIa-V158 by immune complexes (ICs) results in a stronger in vitro cytotoxic potency than that of FcγRIIIa-F158^11^. Regarding the allotype population, FcγRIIIa-V158 homozygotes represent only 10–20% of the population worldwide, whereas FcγRIIIa-F158 homozygotes constitute 40–50%^12,13^. Interestingly, expression of the high-affinity allotype of FcγRIIIa-V158 correlates with the improved clinical outcome in the treatment of lymphoma patients with rituximab^7,10^.

Fc-engineered antibodies with improved affinity to FcγRIIIa show higher therapeutic efficacy relative to native Fc because they are capable of priming and activating NK cells more efficiently^14,15^. However, several studies showed that although the depletion of NK cells or neutrophils did not significantly reduce tumor suppression activity, exhaustion of macrophages abrogated the therapeutic efficacy of anti-CD20^16^, anti-CD30^17^, or anti-CD40 antibodies^18^ in mouse models. These results highlight the importance of ADCP activity in therapeutic efficacy against cancer, because macrophages are one of the main contributing sources of leukocytes for tumor phagocytic activities (Fig. 1b)^19^. ADCP activity is known to be triggered by FcγRIIa intracellular signaling (Fig. 1b)^20–22^, which has been shown by glycoengineered antibodies to exhibit enhanced affinity toward FcγRIIa, resulting in increased ADCP activity^21,23^. However, the existence of other human Fc-glycoengineered antibodies that do not affect FcγRIIa binding^24^ but improve ADCP^20^ also indicates the possibility that FcγRIIIa contributes downstream signaling to phagocytic activity^25^.

### Fcγ receptors II: FcγRIIa and FcγRIIb

FcγRII comprises a single-chain polypeptide α chain with an extracellular region of two immunoglobulin-like domains and a cytoplasmic domain. In humans, FcγRII is further classified into FcγRIIa and FcγRIIb. The activating FcγRIIa (*K*_*D*_ = 2 × 10^−7^ M) contains an ITAM motif in the intracellular region of the α chain, whereas the inhibitory FcγRIIb (*K*_*D*_ *=* 8 × 10^−6^ M) contains an immunoreceptor tyrosine-based inhibitory motif in the same region^26^. FcγRIIa initiates endocytosis, phagocytosis, ADCC, and inflammatory cytokine release. In contrast, FcγRIIb transduces inhibitory signals that downregulate immune functions triggered by the activating receptors. FcγRIIa has two allotypes that differ in a single amino acid at position 131. That residue can be either histidine (H) or arginine (R); the isoform with H is a high-affinity receptor, while that with R is a low-affinity receptor. Despite the high structural homology and protein sequence identity (96%) of FcγRIIa with FcγRIIb^27^, there are a number of ongoing efforts to create engineered antibodies with improved affinity for FcγRIIa to increase the therapeutic potential of the antibody^28–30^.

Several studies have established a correlation between Fc:FcγRs affinities and selectivity and the cytotoxic functions of immune effector cells engaged by ICs. For example, the increased binding toward activating FcγRs over inhibitory FcγRIIb has been shown to be important because it enhances the efficacy of trastuzumab or rituximab^31^. In other studies, the efficacies of various antibodies in the clinic have been shown to correlate with FcγRIIIa alleles (the high-affinity FcγRIIIa-V158 and the low-affinity FcγRIIIa-F158 alleles)^7,10,11^. Additionally, in vitro cytotoxicity was increased with improved affinities for FcγRs either by glycoengineering^32^ or by amino acid substitutions in the Fc domain^33–36^. In particular, Fc variants displaying high affinity for the FcγRIIa-R131 isoform and also high selectivity for FcγRIIa over FcγRIIb have been shown to mediate improved ADCP activity^30^. These results suggest that antibodies with increased binding affinity for activating FcγRs, but not for inhibitory FcγRIIb, elicit stronger ADCP and potentially represent more effective therapeutics for cancer treatment.

### A human IgG recycling receptor: the neonatal Fc receptor (FcRn)

IgG is the most abundant antibody isotype largely because of its extremely long half-life of 7–23 days. The discovery and characterization of an intracellular trafficking receptor for IgG were first reported by Brambell and coworkers in 1966^37^. This receptor is called the neonatal Fc receptor molecule (FcRn) and is a noncovalent heterodimer consisting of an MHC-class-I-like heavy chain and a β2-microglobin light chain. FcRn, which binds to IgG and albumin at different binding sites, is present at the cell surface and intracellularly within the ER.^38,39^ Simister and coworkers first described FcRn as a transporter of IgG within the intestinal epithelial cells of neonatal rats, enabling the transfer of passive immunity from a mother to her offspring^40,41^. Although rodent FcRn is upregulated in intestinal epithelial cells only during lactation, human FcRn is expressed throughout life in almost all other cell types, including endothelial cells^42–44^; kidney podocytes^45^; mammary epithelial cells^46^; pulmonary epithelial cells^47^; hepatocytes^48^; and hematopoietic cells, such as monocytes, macrophages, dendritic cells^49^, and B cells^50^.

Mice deficient in either FcRn or β2-microglobin have severely reduced serum IgG levels because the rates of catabolism of these antibodies are increased. In these mice, IgGs have a circulation half-life of approximately 1.4 days, whereas wild-type mice have a circulation half-life of 9 days^38,51,52^. In humans suffering from familial hypercatabolic hypoproteinemia, a mutation in the β2-microglobin gene results in expression of a nonfunctional protein and five-fold increased catabolism of IgG relative to that in healthy individuals^53,54^. Bone marrow chimeric mouse studies have shown that both wild-type mice chimerized with *Fcgrt−/−* bone marrow and *Fcgrt−/−* mice chimerized with wild-type bone marrow have approximately 50% serum IgG levels relative to those of wild-type mice^55^.

FcRn only binds to its ligands at acidic pH (≤6.5) but not at neutral pH (≥7.0)^56–58^. At physiological pH, histidine residues of the Fc domain of IgG, which interacts with FcRn, become deprotonated, thereby enabling ligand dissociation. In contrast, at more acidic pH, the protonated histidine residues stabilize the IgG:FcRn complex^59^. Thus, the strict pH-dependent ligand-binding property of FcRn seems to have evolved in the endo-lysosomal system to function as a mediator of IgG trafficking and pharmacological parameters, including stability, biodistribution, and immunogenicity, in humans.

## Engineering effector functions of antibodies

The effector functions of antibodies, whether they are produced by our own immune systems or administered therapeutically, rely on the interactions of their Fc domains with FcγRs and complement components, such as C1, to clear pathogens or destroy tumor cells^60–62^. The effector functions of antibodies are: (i) ADCC and ADCP via the recruitment of FcγRs^63^ and (ii) complement-dependent cytotoxicity (CDC) via the recruitment of C1q. The known human FcγRs are FcγRI (CD64), FcγRIIa (CD32a), FcγRIIb (CD32b), FcγRIIc (CD32c), FcγRIIIa (CD16a), and FcγRIIIb (CD16b), and these receptors are expressed at different levels on the surface of various immune cells.^63^ The cytotoxic potential of mAbs results from improved affinity between their Fc domains to the activating FcγR relative to the inhibitory FcγRIIb^31,64^.

NK cells are well-known cytotoxic lymphocytes that kill tumor cells and constitute a major component of the innate immune system. Clinically, NK cells have been implicated as important mediators of the antitumor activity of trastuzumab in breast cancer patients. Patients with responsive tumors tend to have increased numbers of tumor-associated NK cells^65^. ICs are recognized by FcγRIIIa on NK cells, resulting in the activation of cytotoxic processes and potent ADCC^66^. Human NK cells, which only express FcγRIIIa, activate ADCC via FcγRIIIa (Fig. 1a). The efficacy of some cancer therapeutic antibodies has been correlated with a widespread FcγRIIIa polymorphism: improved clinical outcomes are observed in patients expressing the high-affinity isoform V158 rather than the low-affinity isoform F158^7,10,11^.

Optimization of the interactions between antibodies and FcγRs has emerged as a promising approach for enhancing the activity of therapeutic antibodies for the treatment of both cancer and autoimmune disease^67^. Thus, engineering IgGs with improved FcγRs affinity has been investigated extensively in recent years. Researchers at Xencor (CA, USA) capitalized on a structure-based computational design algorithm for engineering various therapeutic effector functions of clinically applicable antibodies. They isolated a series of Fc mutants with improved effector functions mediated by enhanced binding affinity, including increased FcγRIIIa binding over FcγRIIb for ADCC^68^ and increased FcγRIIa binding for ADCP^28^. Remarkably, a S239D/I332E/A330L (EU numbering) IgG mutant showed a > 300-fold increase in FcγRIIIa binding affinity relative to that of the wild-type IgG. This antibody also showed 58-fold increased binding to FcγRIIIa-F158. However, this antibody also bound to the inhibitory receptor FcγRIIb with much higher affinity^68^. For example, margetuximab, developed by MacroGenics, had adapted the same Fab target (Her2) as transtuzumab but had been Fc-engineered to maximize immune effector function by elevating relative affinity to activating Fcγ receptor, FcγRIIIa over inhibitory Fcγ receptor, FcγRIIb, and it recently showed a 24% risk reduction in patients relative to that of trastuzumab in a phase 3 clinical trial in 536 breast cancer patients^69^. Other significant efforts have been directed to the engineering of antibodies with improved affinity for FcγRIIIa and enhanced effector function by amino acid mutations^36,70–74^ or glycan modifications^75–81^. Hatori and coworkers also engineered an antibody Fc variant (P238D/L328E) that showed selectively enhanced FcγRIIb binding over both FcγRIIa-R131 and FcγRIIa-H131^82^. Recently, several engineered variants have shown promise in preclinical testing and are moving into clinical trials^83–85^. These reports indicate the clinical significance of engineered Fcs with improved affinity to FcγRs.

Although antibodies with potentiated tumor-destroying functions of Fcs are desirable for tumor targeting, those with benign immune-silenced Fc’s are advantageous over native Fcs when ADCC, ADCP, and/or CDC would be detrimental, such as when antibodies function as (i) systemic neutralizers of cytokines, (ii) blockers of cell-surface antigens on immune cells, or (iii) bispecific engagers of effector immune cells in proximity to target diseased cells^86–88^. Because it is best not to stimulate unwanted immune cell depletion by FcγRs cross-linking, it is critical to completely silence Fc effector function because of the variation in activation thresholds by FcγRs among patients. One way to reduce the effector function is to remove the *N*-linked glycan by substituting an asparagine residue at 297 position with other residues^89–92^. Another strategy is the incorporation of amino acid mutations into residues that are responsible for FcγRs or C1q binding^93^. For example, Orthoclone OKT3^®^, the first marketed therapeutic antibody, contains two mutations, L234A and L235A, in the Fc domain to minimize toxicity by cytokines^94^. Moving the Fab region to other IgG subclasses, such as IgG2 or IgG4, which engages less tightly to FcγRs could be another option^64^. Moreover, exchanging amino acid residues among IgG subclasses resulted in more Fc options with reduced complement and Fcγ receptor binding, such as IgG2/4^95^, IgG2 m4^96^, and “TM” (L234F, L235E, and P331S)^97^. Researchers at Janssen Research and Development capitalized on multiple methodologies to abolish FcγRs and C1q affinity to generate IgG2σ with undetectable effector functions mediated by FcγRs and C1q^98^. Extensive efforts to engineer Fc to lack effector function are continuously being executed using different approaches, such as proline sandwich^99^, for safety considerations because effector activation thresholds are variable among diseases and populations.

## Engineering serum half-life of antibodies

**Fig. 2:**
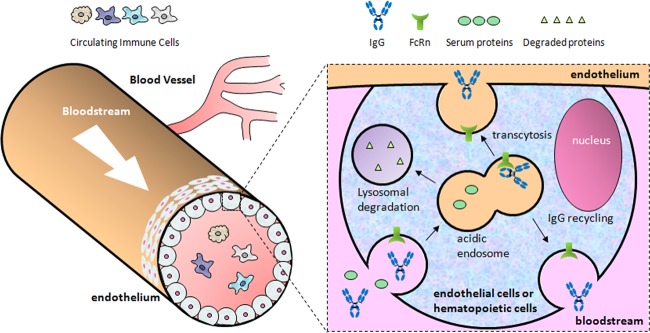
Recycling or transcytosis through endothelium of immunoglobulin G (IgG) antibodies. When IgGs are internalized by an endothelial cell or a circulating monocyte, they initially encounter FcRn within the early acidified endosome. This binding protects IgG from being sorted into a lysosome, in which serum proteins without FcRn binding are degraded; instead, IgGs are recycled back into the extracellular space^44^. At neutral pH on the cell surface, IgGs immediately dissociate from FcRn and return to the circulation.

Compared with other serum proteins in blood circulation, IgG antibodies have the advantage of prolonged serum stability (21 days). FcRn, which exists in endosomes of circulating monocytes or endothelial cells, is a key molecule responsible for controlling serum IgG levels in mammals through its pH-dependent binding characteristics^100^. The mechanism underlying how IgG antibodies can be recycled or transcytosed through endothelium has been established^101,102^ (Fig. 2).

At pH 6.0, FcRn binds to the C_H_2–C_H_3 hinge regions of the Fc region of IgG with micromolar affinity^103,104^. However, it does not measurably bind to IgG at physiological pH. FcRn binds to IgG in acidic endosomal compartments after serum IgG is internalized via pinocytosis by endothelial cells and circulating monocytes (Fig. 2). FcRn then recycles the IgG back into circulation at physiological pH, thus extending its serum half-life, whereas serum proteins that do not bind to FcRn are directed for lysosomal degradation. In contrast to monomeric IgGs that are recycled into serum by FcRn, polymeric IgGs, such as ICs surrounded by multiple IgG molecules bound to antigens, have been shown to be efficiently directed into lysosomes by an unknown mechanism of action^55^. By discriminating IgGs, whether they are monomeric or polymeric, FcRn determines the fate of IgG, either to protect it or direct it to be degraded for antigen presentation purposes. In addition to the enhanced degradation of ICs relative to monomeric IgGs, FcRn has also been shown to contribute to cross-presenting degraded antigens by regulating the sorting of intracellular IgG ICs, thereby cross-priming CD8+ cytotoxic T cell responses^59^.

Extensive studies in non-human primates^105,106^ and in human FcRn transgenic mice^107,108^ have shown that IgG Fc engineering for higher FcRn binding is of great importance for enhancing the half-lives of therapeutic antibodies. Fc engineering for higher FcRn binding at endosomal pH has also been extensively studied. A M252Y/S254T/T256E (YTE) variant isolated by Medimmune using phage display^109^ demonstrated 10-fold greater affinity to human FcRn than that of wild-type IgG1 at pH 6.0 and efficient release at pH 7.4. IgG antibodies containing YTE mutations showed 3.8-fold improvement in pharmacokinetics (half-life) in human FcRn transgenic mice and 2.5-fold improvement in cynomolgus monkeys^105,110,111^. However, YTE exhibited decreased ADCC activity. The Medimmune YTE had been adapted to anti-RSV antibody Fc, which received fast track designation by the FDA for prevention of respiratory disease under the name of MEDI8897, and is currently undergoing phase IIb clinical evaluation^111^. Another corporate group, Xencor, developed an engineered variant, Xtend (M428L/N434S), with 11-fold increased FcRn binding at pH 6.0 relative to that of wild-type IgG1. Xtend showed 4.2-fold improved serum half-life in transgenic mice and 3.2-fold improved serum half-life in non-human primates. Xtend Fc was tested in xenograft mouse models that express human FcRn as either an anti-VEGF or anti-EGFR IgG1 antibody and led to extended serum half-life as well as reduced tumor burden relative to those of wild-type IgG1^108^. Xtend has been adapted to ravulizumab (ALXN1210), which has been approved by US FDA on December 2018 for treatment of paroxysmal nocturnal hemoglobinuria/hemolytic-uremic syndrome^112^, or VRC01LS, which is under clinical evaluation for the prevention of human immunodeficiency virus^113^. These studies indicate that prolonging the serum stability of engineered IgG antibodies in the blood circulation enables extended therapeutic or protective activity, which circumvents the need for frequent administration of antibody drugs.

In some cases, the extended half-life property of Fc is desirable when combined with “benign blockers” with abolished effector functions, which are described in the section outlining engineering effector functions of antibodies. Borrok et al. at Medimmune characterized the molecular stability of a combination of two variants: “TM” (L234F, L235E, and P331S), which lacks immune receptor binding^97^ and “YTE” (M252Y, S254T, and T256E), which has an extended serum half-life^105^. Furthermore, they dissected the contributions of individual mutations of TM-YTE, which in turn hamper thermostability, and then discovered a novel combination FQQ-YTE (L234F, L235Q, K322Q, M252T, S254T, and T256E), with improved molecular and serum stability, which capitalized on rational protein engineering design^114^.

## Engineering valency or bispecificity of antibodies

Fc engineering efforts for better therapeutic efficacy, other than for modulating affinity profiles to Fc receptors, are also being extensively pursued. One example is an engineered Fc that does not form a homodimer but remains as a soluble monomer, mFc, with half size, displaying (i) high affinity for FcγRI, (ii) no detectable binding to FcγRIIIa, and (iii) similar pH-dependent FcRn binding^115,116^. This engineering was focused on downsizing the molecular weight of Fc for therapeutic simplicity. However, mFc could nonspecifically bind to several viral or cancer-related proteins, such as gp140 of HIV-1, EDII of Zika virus, mesothelin, 5T4, PD-L1, OX40, and TIM3. Nonspecific bindings have been excluded by phage-display library screening, and the critical role of T366R and L368H on building monomeric status has been identified^117^.

Fc engineering for heterodimeric Fc is another example of obtaining bispecific properties for antigen binding to circumvent homodimer formation. The bispecific antibodies recognize two different antigens, so that they not only neutralize soluble pathogenic or endogenous proteins, but also co-ligate discrete antigens on the same cells or engage unconnected cells to derive therapeutically favorable status. Until now, three strategic approaches have been adapted to achieve heterodimeric structure in the Fc domain: (i) steric complementarity design, which are generally called knobs-into-holes; (ii) charge-to-charge swap; and (iii) isotype strand swap^118–120^. Two bispecific mAbs are currently on the market: emicizumab (Hemlibra^®^) and blinatumomab (Blincyto^®^). Emicizumab, developed by Chugai Pharmaceutical, is an IgG4 knobs-into-holes bispecific for coagulation factors IX and X to mimic coagulation factor VIII (FVIII) activity and is more favorable than FVIII in terms of pharmacokinetics (21 days for emicizumab vs. 0.5 day for FVIII)^121^. The Fc-engineered bispecific IgG4 antibody, emicizumab has been approved by the FDA for prophylaxis to prevent or reduce the frequency of bleeding episodes in patients with hemophilia A on November 2017^122–124^. This Fc engineering for bispecificity enabled co-ligation of two different antigens and opened up new therapeutic opportunities, maintaining all of the beneficial properties of mAbs.

The other marketed bispecific antibody is blinatumomab, a fusion of the two single-chain Fvs targeting CD3 on T lymphocytes and CD19 on tumor cells, which was approved by the FDA in December 2014. Despite their significant efficacy, the current bispecific T-cell engager (BiTE) programs have serum half-lives of <1 day, and therefore require continuous intravenous infusion for 28 consecutive days per cycle. Hence, Amgen is currently advancing new versions of BiTE molecules by fusing the Fc domain. However, the fact that catumaxomab, a bispecific mAb for CD3, and EpCAM have been withdrawn due to toxicity issues, presumably because of FcγR or C1q binding of Fc^125–128^, indicates that for T-cell engagers, FcγR-related effector functions are detrimental with respect to clinical aspects. Therefore, simple Fc fusion for half-life extension purposes may lead to side effects, such as toxicity and/or shortened efficacy, at least in case of BiTE. Either Fc engineering for immune silencing or further protein engineering are also essential for immune cell-engaging antibodies.

## Conclusion

Extensive research and development capitalizing on Fc engineering techniques to modulate effector functions of antibodies have been conducted by researchers in both academia and industry^129^. Despite these efforts, the exact function of Fc remains to be validated; for example, the function of FcγRIIIa on NK cells is the key contributor for ADCC activity but that on other lymphocytes, such as monocytes and macrophages, is not yet clear. Especially, Fc function in protection of infectious diseases, such as antiviral activity, has received increasing attention^130,131^, which suggests that it is responsible for inducing humoral and cellular responses that provide protective immunity, such as vaccine-like effects, in addition to neutralizing the activity of IgG antibodies. Fc engineers have also developed a few Fc variants that have stricter pH-dependent FcRn binding, such as YTE and Xtend, which are used in clinical settings as anti-infectious agents or against autoimmune diseases^132^. The recent approvals of bispecific antibodies, developed by using sophisticated Fc engineering that produced maximal ratio of heterodimeric Fcs relative to homodimeric Fcs, indicate that bispecific antibodies have high potential for therapeutic applications and that there is much room for improvement to meet unmet medical needs, such as in pharmacokinetics or tissue infiltration, while maintaining low toxicity. Despite insufficient knowledge of the functions of Fc receptors because of the diverse profiles of the receptors on discrete leukocytes, Fc engineering works for (i) improving effector functions by selective FcγR affinity in tumor targeting purposes, (ii) maximizing serum half-life by pH-selective FcRn affinity for various disease categories such as cancer, infectious diseases or autoimmune therapy, (iii) molecular downsizing to half relative to that of native antibodies by blocking homodimerization, and (iv) conferring bispecificity by maximizing the efficiency of Fc heterodimerization. These are powerful strategies to broaden the therapeutic applications of antibodies. Additionally, ICs comprising antibodies that have had their Fc functions optimized for adaptive immunity are potential candidates for future cancer vaccines and therapy^133,134^.
